# Navigating the Complexities of Molar Incisor Hypomineralization: Challenges and Strategies in Pediatric Dentistry

**DOI:** 10.1155/ijod/9329492

**Published:** 2025-01-07

**Authors:** Zuhair Al-Nerabieah, Muaaz AlKhouli, Mayssoon Dashash

**Affiliations:** Pediatric Dentistry Department, Faculty of Dentistry, Damascus University, Damascus, Syria

**Keywords:** children, management, molar incisor hypomineralization, pediatric dentistry

## Abstract

**Background:** Molar incisor hypomineralization (MIH) presents a multifaceted challenge in pediatric dentistry, impacting diagnostics, clinical management, resource accessibility, and psychosocial care. The condition's complexity is exacerbated by diagnostic variability, overlapping clinical symptoms, and the need for tailored treatment approaches.

**Objectives:** This study aims to explore the key challenges associated with the management of MIH in pediatric dentistry, including diagnostic precision, clinical management, resource limitations, interdisciplinary care, long-term follow-up, and psychosocial impact, and to propose strategies for overcoming these obstacles.

**Methods:** A comprehensive literature review was conducted to identify and synthesize existing evidence on MIH's etiology, diagnosis, treatment, and prognosis. The review highlighted the barriers encountered in providing optimal care, particularly in resource-constrained settings, and explored potential solutions through clinical and interdisciplinary approaches.

**Results:** The key findings included the need for standardized diagnostic criteria, the role of individualized treatment plans, and the importance of resource allocation. Limited access to specialized equipment and education hampers care, especially in under-resourced areas. Long-term management complexities were further compounded by the necessity for interdisciplinary collaboration and attention to psychosocial factors affecting pediatric patients.

**Conclusion:** Effective MIH management requires standardized diagnostic protocols, resource advocacy, interdisciplinary collaboration, and holistic patient care. Advancements in research, education, and policy are essential to improve outcomes in pediatric patients. By addressing both clinical and psychosocial challenges, the overall well-being of MIH-affected children can be enhanced.

## 1. Introduction

Molar incisor hypomineralization (MIH) stands as a significant and complicated challenge within the realm of pediatric dentistry, profoundly influencing the oral health landscape for affected children [1]. MIH, characterized by hypomineralized enamel in the first permanent molars and often accompanied by affected incisors, has emerged as a prevalent dental anomaly with notable implications [2, 3]. As we embark on this exploration, it becomes imperative to delve into the multifaceted aspects of MIH, understanding its prevalence, clinical manifestations, and the ensuing challenges that pediatric dentists encounter in its diagnosis and management.

MIH, first officially recognized in the early twenty-first century, has garnered increasing attention due to its impact on the developing dentition of children. The condition disrupts the normal mineralization process during tooth development, resulting in enamel that is structurally compromised and more susceptible to breakdown and dental caries [4, 5]. While the exact etiology remains elusive, a combination of genetic and environmental factors is believed to contribute to its manifestation, further underscoring the complexity of this dental anomaly [6].

Prevalence studies have shown MIH affecting a noteworthy proportion of the global pediatric population, with regional variations observed [7]. This variability necessitates a comprehensive understanding of the epidemiology, aiding in the development of targeted diagnostic and management strategies. Additionally, the condition's prevalence underscores its clinical relevance, demanding attention not only from dental practitioners but also from the broader healthcare community [8].

The present research seeks to unravel the challenges inherent in managing MIH in pediatric dentistry, recognizing the significance of addressing these intricacies for the betterment of patient outcomes. Pediatric dentists, as primary custodians of children's oral health, are confronted with a myriad of challenges in the diagnosis, treatment planning, and long-term management of MIH [3]. The importance of this study lies in its potential to shed light on these challenges, offering insights that can inform evidence-based practices and guide future research endeavors.

By navigating the complexities of MIH, this review aimed to identify and examine the primary challenges in managing MIH, focusing on the areas of etiology, diagnosis, treatment, and prognosis.

## 2. Search Strategy and Study Selection

### 2.1. Search Strategy

A comprehensive search strategy was employed to explore the challenges associated with managing MIH in pediatric dentistry. The Population, Intervention, Outcome (PIO) framework was used to guide the search, with a focus on four key domains: etiology, diagnosis, treatment, and prognosis:

• Population/Problem (P): Pediatric patients with MIH
• Intervention/Issue (I): Etiology, diagnosis, treatment, and prognosis
• Outcome (O): Challenges in MIH management

The review focused on articles published from 2013 to 2023 to ensure relevance to modern clinical practices. Inclusion criteria comprised peer-reviewed studies in English that specifically addressed key aspects of MIH in pediatric patients. Articles were selected if they provided insights into one or more of the following areas: etiological factors, diagnostic methods, treatment modalities, or long-term outcomes relevant to MIH management. We included studies focusing on children or adolescents, aiming to capture findings most applicable to pediatric dentistry.

Exclusion criteria were applied to studies that did not focus on pediatric populations, lacked specific emphasis on MIH, or did not address MIH-related clinical challenges. Additionally, articles were excluded if they did not offer relevant insights into the unique challenges or management strategies associated with MIH in pediatric cases, including studies limited to adult populations or those focusing on broader dental hypomineralization without MIH-specific data.

### 2.2. Database Search

The primary database used was PubMed, with additional searches conducted on Google Scholar and Scopus to ensure comprehensive literature coverage. The search strings combined Medical Subject Headings (MeSH) terms with keywords, ensuring a thorough exploration of relevant literature.

Boolean operators (AND/OR) were used to refine the search results and capture studies across the domains of etiology, diagnosis, treatment, and prognosis.  Table 1 provides a detailed overview of the search strings and criteria used in this review.

### 2.3. Study Selection Process

The initial search yielded a total of 342 articles. Two independent reviewers conducted a thorough screening of titles and abstracts to assess relevance. Articles passing the initial screening were evaluated in full text to determine eligibility based on the predefined inclusion and exclusion criteria. Any disagreements between the reviewers were resolved through discussion or, when necessary, by consulting a third reviewer.

### 2.4. Categorization of Challenges

**Figure 1:**
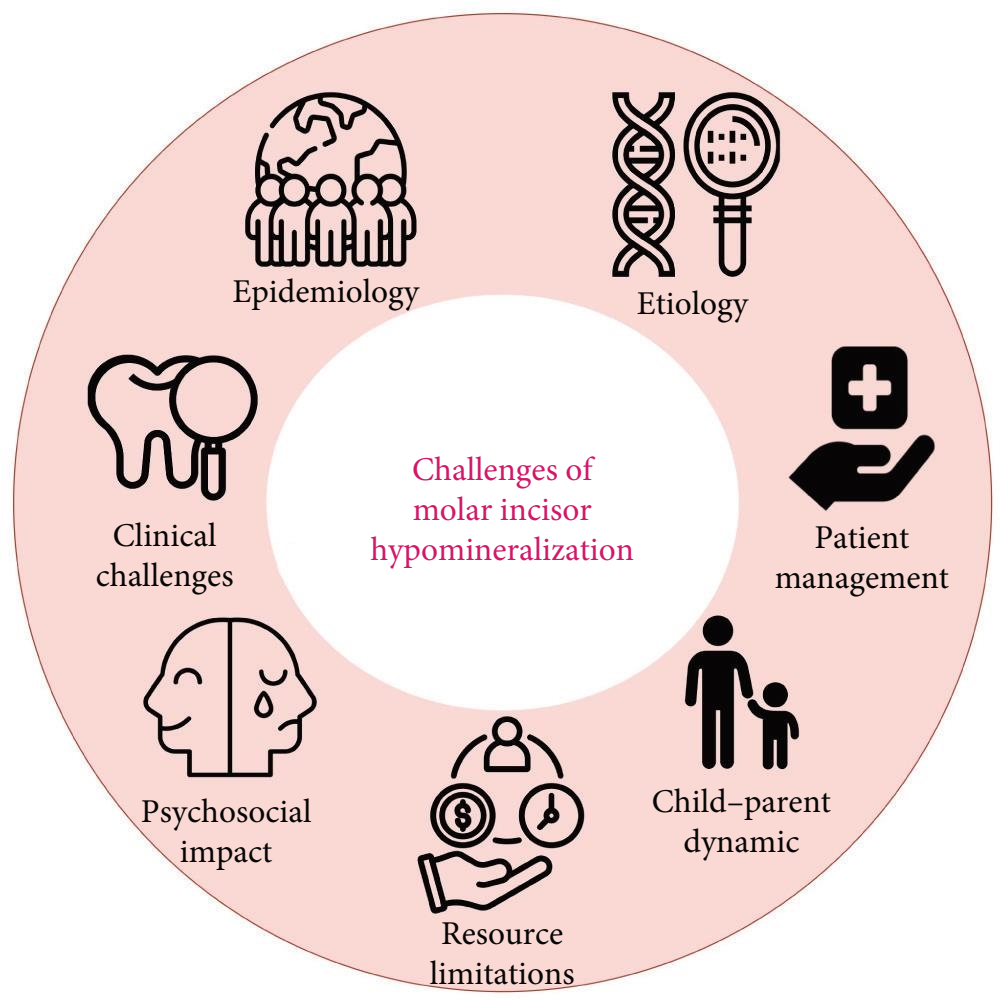
Challenges in the management of molar incisor hypomineralization.

Based on the selected studies, the challenges associated with MIH management were categorized into the following key domains (Figure 1):

• Epidemiology and diagnosis
• Etiology and risk factors
• Clinical manifestations
• Psychosocial impact
• Patient management
• Child–parent dynamics
• Resource limitations

### 2.5. Summarization and Discussion

**Figure 2:**
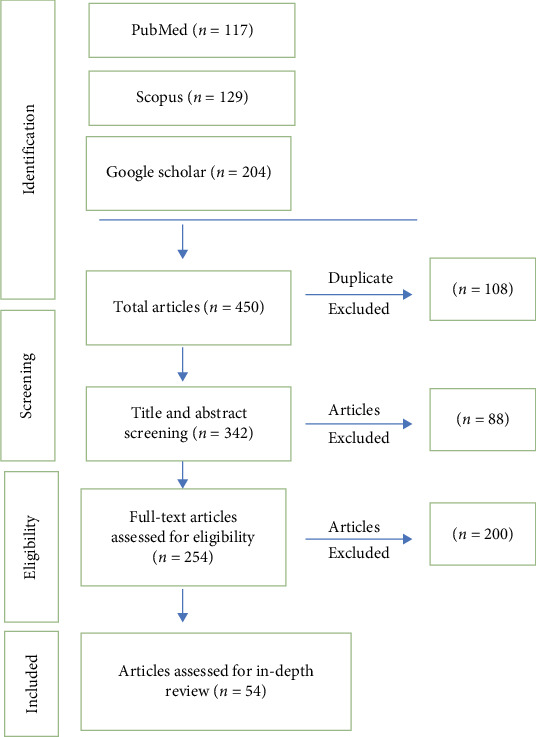
PRISMA flowchart for search strategy.

Out of the 342 articles initially identified, 54 studies were selected for in-depth review. A narrative synthesis approach was used to summarize the findings, allowing for a comprehensive discussion of the multifaceted challenges in MIH management and their implications for pediatric dentistry. This method not only highlighted the challenges but also provided a deeper understanding of their complexity and potential solutions (Figure 2).

## 3. Epidemiology and Diagnosis

### 3.1. Prevalence Rates

MIH has emerged as a prevalent concern in pediatric dentistry, impacting the global oral health landscape with varying degrees of occurrence [9]. Extensive epidemiological studies conducted across diverse populations have revealed prevalence rates ranging from 2% to 40%, underlining the considerable variability in its manifestation worldwide [10, 11]. Furthermore, the frequency of MIH reported in the Middle East region ranged from 2.3% to 40.7% [12, 13]. However, a recent systematic review has estimated the mean global prevalence of MIH at 13.5% [14]. This wide prevalence spectrum necessitates a significant understanding of regional and demographic factors that may influence the incidence of MIH (Table 2).

Several factors contribute to this variability, including geographic location, socioeconomic status, and even cultural practices [18]. The identification of such variations is critical for tailoring diagnostic and management approaches to specific demographic profiles, thereby ensuring targeted and effective interventions.

Prevalence studies on MIH face multiple challenges, complicating efforts to obtain reliable data. One primary challenge is the lack of standardized diagnostic criteria, leading to inconsistent definitions and variability in diagnosing MIH across studies. Differences in examiner experience and training can further affect the diagnosis, as MIH is often visually assessed, which may result in underreporting or overreporting [18].

Geographic variability is another significant factor; prevalence can differ widely between regions, likely due to environmental, genetic, and socioeconomic factors, making it difficult to generalize findings. Variability in the age groups studied also affects prevalence reporting, as MIH tends to manifest differently depending on the age at which children are assessed [10, 11].

This can cause inconsistencies in prevalence rates, as younger children may not yet display all MIH features, while older children may have experienced restorations or extractions due to severe lesions. Finally, differences in sampling methods—whether studies are conducted in clinical, school, or community settings—further contribute to variability in reported prevalence, impacting the generalizability of findings across populations. Together, these challenges highlight the need for standardized diagnostic protocols and consistent study designs to better understand the true prevalence of MIH [13].

Moreover, the potential for misdiagnosis of MIH remains a significant concern, particularly due to a lack of experience among general practitioners. Unlike pediatric dentists who are more acquainted with the intricacies of MIH, general practitioners may encounter challenges in confidently identifying the condition. This highlights the importance of specialized training and increased exposure to MIH cases to enhance diagnostic accuracy, ensuring that the prevalence and impact of MIH are not underestimated in epidemiological studies led by clinicians with varied levels of experience [19].

### 3.2. Diagnostic Challenges

Despite advances in dental diagnostic methodologies, the early identification of MIH remains a formidable challenge for dentists [15]. The clinical presentation of MIH can mimic other developmental dental anomalies, such as enamel hypoplasia or fluorosis, leading to diagnostic confounders [20]. Furthermore, distinguishing MIH from common dental conditions like dental caries poses additional hurdles, particularly in the early stages when subtle enamel defects may be less apparent [3].

One of the primary diagnostic challenges lies in the variability of MIH presentations, ranging from mild opacities to severe enamel breakdown. This diversity in clinical manifestations complicates the establishment of standardized diagnostic criteria and requires clinicians to exercise a high degree of clinical judgment. Additionally, the condition's predilection for affecting the first permanent molars and incisors, crucial teeth in the early dentition, intensifies the urgency for accurate and timely diagnosis [2, 16].

Diagnostic modalities such as visual–tactile examination and radiographic analysis are essential tools in the diagnosis of MIH. However, these traditional methods have limitations, often requiring a more comprehensive and multidimensional approach to ensure accurate diagnosis. While establishing standardized diagnostic criteria remains an ongoing challenge, emerging technologies are showing promise in improving diagnostic precision [16].

One such advancement is the use of reveal fluorescence dental loupes, which utilize fluorescence technology to enhance the visual detection of hypomineralized enamel. This tool allows clinicians to differentiate MIH lesions more clearly from other dental anomalies, such as enamel hypoplasia or early caries, facilitating more accurate early-stage diagnosis [21, 22]. By improving visualization of subtle enamel defects, fluorescence technology offers a valuable adjunct to the conventional diagnostic process, aiding pediatric dentists in identifying MIH with greater accuracy and consistency [23].

The epidemiological diversity and diagnostic intricacies associated with MIH underscore the imperative for ongoing research efforts to refine diagnostic criteria, enhance clinician training, and develop innovative diagnostic tools. Addressing these challenges is paramount in ensuring early detection and, subsequently, effective management of MIH in the pediatric dental setting.

## 4. Etiology and Risk Factors

### 4.1. Genetic and Environmental Factors

The potential genetic basis of MIH has gained significant attention, with evidence from family studies indicating a hereditary component. A higher prevalence of MIH among siblings and monozygotic twins suggests a genetic predisposition, although the specific genetic mechanisms remain under investigation [24, 25]. Researchers are exploring polymorphisms in genes related to enamel formation, mineralization, and amelogenesis, pointing to a possible role of these genetic variants in MIH development [26, 27]

Environmental factors are also key contributors to the etiology of MIH, often interacting with genetic susceptibilities [28]. Perinatal factors such as hypoxia, caesarean section, and prematurity, along with postnatal conditions like bronchitis, pneumonia, and asthma, have been associated with a heightened risk of MIH [23, 29].

Furthermore, a systematic review by Garot et al. [23] highlighted that peri- and postnatal etiological factors are more likely to increase the odds of causing MIH than prenatal factors. This finding emphasizes the importance of focusing on perinatal and postnatal health conditions when investigating the origins of MIH [23].

Additionally, fever and antibiotic use, often consequences of infant and childhood illnesses, exhibit correlations with the occurrence of MIH. Notably, some factors previously suggested as potential contributors to MIH, including prolonged breastfeeding, low birth weight, and maternal habits such as smoking, alcohol consumption, or medication use, do not exhibit significant associations with the onset of MIH [23].

Moreover, emerging research highlights a potential genetic component to MIH, with studies indicating familial aggregation patterns that suggest heritability. Some research proposes the involvement of gene–environment interactions, where genetic predispositions may heighten susceptibility to environmental stressors affecting enamel formation. Recently, the role of oxidative stress and systemic inflammation has gained attention as contributing factors, further expanding the understanding of the condition's etiology [27].

### 4.2. Association With Systemic Conditions

MIH's potential links to systemic health have added further complexity to its etiological investigation. While primarily viewed as a dental condition, MIH has been associated with a range of systemic conditions, including respiratory infections, febrile illnesses, and other childhood diseases [30]. These associations suggest that systemic health factors during critical periods of enamel development may influence MIH manifestation [31]. Additionally, prenatal and perinatal events have been implicated in some studies, reinforcing the idea that systemic health plays a role in enamel development [23].

The growing recognition of MIH as a potential marker of broader systemic health issues emphasizes the need for a holistic approach to its management. By understanding these systemic associations, we can better address MIH not just as a dental condition but as part of a broader pediatric health context [26].

The etiology of MIH is driven by a complex interaction of genetic, environmental, and systemic factors. Ongoing research into these areas offers promising pathways for the development of targeted preventive and therapeutic strategies, aimed at mitigating the impact of MIH in pediatric populations.

## 5. Clinical Manifestations

### 5.1. Beyond First Permanent Molars and Incisors

MIH exhibits a spectrum of clinical manifestations that extend beyond its primary impact on the first permanent molars and incisors. While these teeth are commonly affected, it is important to recognize that MIH can also involve other tooth types, such as second permanent molars, premolars, and canines [16]. The presence of hypomineralization in these additional teeth adds complexity to the diagnosis, requiring a thorough examination of the entire dentition.

Among the affected teeth, second permanent molars stand out as significantly susceptible to MIH-related hypomineralization. Patients exhibiting severe MIH in their first permanent molars often display a higher frequency of mild defects in their second permanent molars. This correlation suggests a potential pattern of enamel disruption that extends beyond the initially affected teeth [32]. The presence of mild defects in the second permanent molars serves as an intriguing aspect of the clinical manifestations of MIH, offering insights into the systemic nature of this dental anomaly. Understanding these interrelationships provides valuable information for clinicians, aiding in both the diagnosis and comprehensive management of MIH in pediatric patients.

Moreover, the early identification of MIH-related problems in the second primary molars can serve as a predictive marker [10, 33]. Despite the limitations inherent in the published studies, the presence of hypomineralized second primary molars (HSPM) emerges as a predictive factor MIH. Recent study has indicated a noteworthy association between the presence of mild HSPM and a higher prevalence of MIH. This finding highlights the potential significance of HSPM as a clinical indicator, offering practitioners a valuable tool for early identification and risk assessment of MIH in pediatric patients [33].

While acknowledging the study's limitations, including the need for further research to validate these findings, the observed link between HSPM and MIH adds depth to our understanding of the predictive factors associated with this complex dental condition. It further highlights the need for timely intervention and tailored treatment strategies to address the diverse clinical manifestations of MIH, promoting optimal oral health outcomes in affected individuals.

### 5.2. Clinical Appearance and Classification of MIH

The clinical presentation of MIH is highly variable, with manifestations ranging from mild to severe, making accurate classification essential for diagnosis and treatment planning. MIH primarily affects the first permanent molars and incisors, though other teeth may also be involved. The hallmark clinical feature is enamel hypomineralization, which can appear as well-demarcated opacities in shades of white, yellow, or brown, depending on the severity of the lesion [3, 34].

In mild cases, MIH may present as slight opacities with no enamel breakdown, often going unnoticed during routine dental exams. However, in more severe cases, the enamel can be fragile and prone to rapid breakdown under masticatory forces, leading to posteruptive enamel loss and increased susceptibility to caries [35]. The presence of these defects not only compromises the esthetics of the affected teeth but also leads to functional difficulties such as tooth sensitivity and pain, especially in response to thermal stimuli. This discomfort can severely impact the patient's quality of life, particularly during eating or toothbrushing [36].

**Figure 3:**
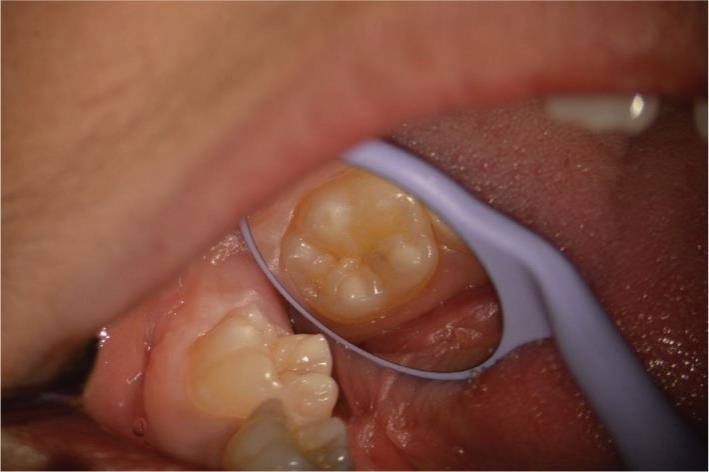
First permanent mandibular molar affected by mild molar incisor hypomineralization (MIH).

**Figure 4:**
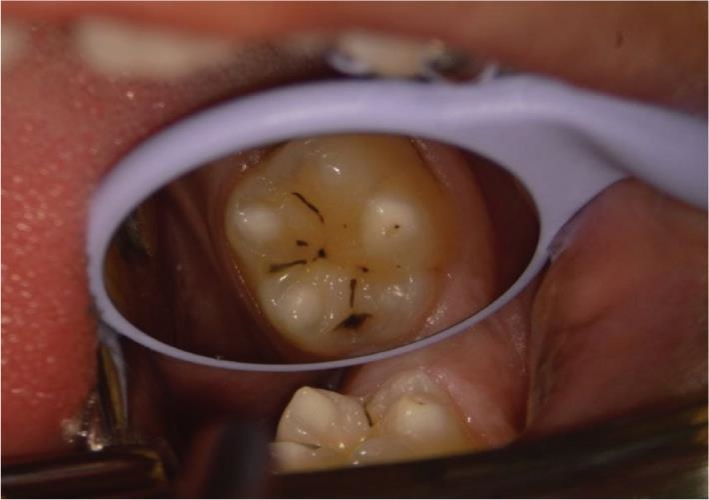
First permanent mandibular molar affected by severe molar incisor hypomineralization (MIH).

Classification systems for MIH help clinicians evaluate the severity of the condition and determine appropriate treatment options. Common classifications categorize MIH lesions based on their color, size, and the presence of posteruptive breakdown. For example, mild MIH is characterized by small, whitish yellow opacities with intact enamel, while severe MIH presents with large opaque/brownish opacities, enamel breakdown, dental caries, and significant sensitivity or pain [35] (Figures 3 and 4).

Accurately diagnosing MIH requires differentiating it from other developmental enamel defects, such as dental fluorosis or enamel hypoplasia. The distinct, well-demarcated nature of MIH lesions, in contrast to the more diffuse appearance of fluorosis, aids in this differentiation. However, overlapping features can still complicate the diagnostic process, requiring a detailed examination of each affected tooth [34].

By focusing on the clinical appearance and employing a structured classification system, dental practitioners can better diagnose MIH, differentiate it from similar conditions, and tailor their treatment strategies based on the severity of the condition. Accurate classification is crucial to guiding both preventive and restorative interventions, ultimately improving patient outcomes.

## 6. Psychosocial Impact

MIH extends beyond its physiological implications, influencing the psychosocial well-being of pediatric patients [37]. This can lead to feelings of self-consciousness, embarrassment, and dissatisfaction, which may negatively impact a child's self-esteem and social interactions [38]. Children with MIH often face teasing or bullying, which can contribute to anxiety, social withdrawal, or reluctance to participate in social activities [39, 40]. Pediatric patients may struggle to express these emotional challenges, requiring sensitive communication between dental professionals, parents, and the child to effectively address psychosocial concerns [39, 40].

The psychosocial impact of MIH extends to oral health-related quality of life, influencing emotional well-being and daily activities. This multifaceted impact requires a holistic care approach, which includes addressing parental anxiety. Open communication and collaborative decision-making help balance clinical interventions with parental concerns [41]. Failure to address the psychosocial aspects of MIH can lead to long-term psychological effects, emphasizing the need for ongoing support [42].

Cultural factors also shape perceptions of dental esthetics and influence the psychosocial burden of MIH. In cultures where appearance plays a key role in social status or personal hygiene, children with visible MIH lesions may experience heightened anxiety or social challenges. Conversely, some cultures may prioritize functionality over esthetics. Understanding these cultural nuances is crucial for dental professionals to provide personalized care that aligns with the values and expectations of the family, improving psychosocial support outcomes [39, 40].

A holistic, patient-centered approach that integrates effective communication, cultural sensitivity, and collaboration with mental health professionals can greatly enhance the overall well-being of pediatric patients with MIH [42].

## 7. Patient Management

### 7.1. Treatment Planning

Effectively managing MIH in pediatric patients demands a judicious approach to treatment planning, taking into account the unique challenges posed by the condition, the child's age, compliance, and long-term oral health goals [16].

An individualized treatment approach stands as a cornerstone in addressing MIH, given the spectrum of severity exhibited by the condition. Treatment plans must be meticulously tailored to meet the specific needs of each patient, considering factors such as the extent of enamel defects, the affected teeth, and the overall oral health status. This individualization ensures a targeted and patient-centred strategy, optimizing outcomes for diverse presentations of MIH [36].

Managing MIH effectively requires an adaptable treatment strategy that accounts for the variable severity of the condition, the patient's age, and individual needs. Based on the Würzburg concept, the foundation of treatment involves prophylactic measures. This includes the use of high-fluoride toothpaste (1450 ppm), fluoride varnish applied two to four times a year, and casein phosphopeptide-amorphous calcium phosphate (CPP-ACP) pastes for patients with severe cases, to enhance remineralization and reduce sensitivity [43].

In noninvasive therapy, fissure sealants are commonly recommended for erupting molars, while glass ionomer cement (GIC) may be used temporarily for partially erupted teeth. For anterior teeth with mild defects, esthetic approaches such as resin infiltration, microabrasion, and tooth whitening are gaining popularity, as they can mask opacities without significant removal of enamel [36, 43].

For moderate to severe cases, minimally invasive techniques like silver diamine fluoride (SDF) offer an alternative for arresting caries, especially in younger children or uncooperative patients. SDF's antibacterial properties provide long-term caries prevention, though its esthetic drawbacks (tooth staining) can limit its use in visible areas [44, 45]. Temporary restorations, including glass ionomer and resin-modified glass ionomer, offer relief for hypersensitive teeth while protecting enamel until definitive care is feasible [37].

In more severe cases involving extensive enamel breakdown, definitive permanent restorations such as composite resin or full-coverage restorations like stainless steel or zirconia crowns are recommended, particularly for molars with structural damage. These crowns offer long-term durability and can withstand occlusal forces in pediatric patients. The advent of bioactive materials, such as bioactive glass and calcium silicate cements, is also promising for MIH management, as they promote remineralization and reduce sensitivity while maintaining tooth structure [16, 43].

Bonding restorative materials to MIH-affected teeth presents significant challenges due to the altered enamel and dentin structure characteristic of this condition. Enamel affected by MIH is often porous, hypomineralized, and less dense, making it more susceptible to fracture and degradation during preparation and adhesive procedures [16]. The high porosity and lower mineral content of MIH enamel reduce its etching quality, leading to compromised bonding strength with conventional adhesives. Additionally, the presence of surface irregularities and protein-rich enamel hampers the penetration of bonding agents, potentially reducing the longevity of restorations [26].

In addition to compromised enamel, dentin in MIH-affected teeth may also exhibit structural deficiencies, further complicating adhesive performance. The hypersensitivity often experienced by MIH patients can make moisture control difficult during bonding, which is critical for effective adhesion. Studies have shown that pretreatment strategies, such as selective etching or the use of resin infiltrants, can sometimes improve bonding efficacy, although these techniques are not universally successful and require careful technique to avoid damaging the fragile enamel further [36]. Alternative approaches, including GIC or bioactive materials, are sometimes favored for their chemical bonding ability and fluoride release, offering improved adaptation and protection for compromised MIH teeth. However, these materials may not achieve the same level of esthetics and durability as composite resins, posing an additional clinical challenge in the long-term management of MIH-affected dentition [43].

For severely affected teeth with pulp involvement, endodontic treatment or extraction becomes necessary, with orthodontic planning integrated into the decision-making process to avoid malocclusion. Delaying extraction can sometimes worsen the prognosis, so timely intervention is key [43].

Finally, long-term monitoring and reassessment of treatment are crucial. As the child grows, treatment plans must evolve to address new challenges, such as secondary caries or orthodontic needs [35].

This comprehensive and individualized approach not only addresses the physical defects associated with MIH but also ensures the psychosocial well-being of pediatric patients, fostering better cooperation and long-term oral health outcomes. New treatment modalities, such as bioactive materials and the use of SDF, along with the incorporation of digital dentistry technologies for precise restoration planning, enhance the effectiveness of MIH management.

### 7.2. Pain Management

Pain and anxiety represent common challenges associated with MIH, introducing additional considerations for pediatric dentists in managing patient comfort [46]. Furthermore, hypersensitivity emerges as a significant challenge in the realm of MIH, representing a crucial symptom alongside posteruptive enamel breakdown. Chronic pain sensations often manifest early, particularly during the eruption of molars, potentially attributed to the infiltration of oral bacteria through hypomineralized enamel into dentinal tubules. This phenomenon, even in the absence of enamel breakdown, underlines the importance of thorough clinical examination and regular monitoring of MIH-affected teeth [47].

Children grappling with MIH-related hypersensitivity face a dual deficit, characterized by acute and chronic pain. Acute hypersensitivity can lead to compromised oral hygiene, difficulties with consuming hot and cold foods, and increased susceptibility to caries due to restricted hygiene practices. In severe cases, this may trigger destructive spirals, hastening the deterioration of affected teeth. Chronic hypersensitivity, on the other hand, contributes to pain–anxiety conditions, limiting the child's cooperation with dental procedures and fostering a pain memory that may intensify pain sensations even without direct stimuli [48].

Managing pain in MIH patients requires a complicated approach, acknowledging the psychological impact on children. Anxiety, limited cooperation, and the development of pain memory are common in those with hypersensitive MIH teeth. Child-friendly treatment protocols, incorporating fear-avoidance conversations, the tell–show–do technique, conditioning, positive reinforcement, distraction, attention control, systematic desensitization, and cognitive modeling, form the foundation for successful dental interventions [3, 48].

In the context of hypersensitivity, especially in severely hypoplastic first molars, achieving painless dental treatment proves challenging. Interruptions during treatment sessions and ineffective anesthesia are reported issues, with studies indicating a correlation between the severity of hypersensitivity and the quality of restoration. Chronic pulp inflammation, stemming from porous hypomineralized enamel and dentin layers, appears as a key contributor to hypersensitivity, predominantly affecting first permanent molars [49].

Anesthesia in MIH-affected teeth is often less effective, posing a unique challenge during dental procedures. The altered enamel and dentin structure associated with MIH, including increased porosity and reduced mineral content, can result in hypersensitivity, making patients more reactive to stimuli. This sensitivity is believed to arise from the extensive porosity and irregularities within the enamel, allowing external stimuli to penetrate more easily to the pulp. Additionally, MIH-affected teeth are often hyperemic, with inflamed pulpal tissues that can lower the pain threshold, further complicating anesthetic effectiveness. Hence, supplemental techniques, such as intraligamentary or intraosseous injections, are sometimes necessary to provide effective anesthesia [16].

To address the unique challenges posed by MIH-related hypersensitivity, a comprehensive pain management strategy is essential. Painless examinations, confidence-building measures, systematic intraoperative pain control through local anesthesia, premedication when necessary, postoperative pain control, additional sedation for deeply fearful children, and, in severe cases, general anesthesia for major interventions form the pillars of a holistic approach [50].

Strict adherence to these principles, coupled with a tailored treatment protocol based on the MIH-Treatment Need Index (MIH-TNI), ensures optimal pain control and behavior management for MIH patients requiring restorative interventions [43]. Pediatric dentists must be attuned to the increased sensitivity, anxiety, and secondary caries risk in MIH patients, avoiding blame attribution to parents and prioritizing a compassionate and effective treatment approach.

## 8. Child–Parent Dynamics in MIH Care

### 8.1. Patient Cooperation

Implementing treatment strategies for MIH relies heavily on pediatric patient cooperation, presenting distinct challenges that necessitate careful consideration [16].

Tailoring communication strategies to the child's age and developmental stage is paramount for effective engagement in preventive measures. Younger children may have limited comprehension, requiring simplified explanations and visual aids to convey the importance of preventive practices. This age-appropriate communication fosters understanding and cooperation [48].

Behavioral management techniques, including positive reinforcement and distraction, play a pivotal role in fostering cooperation during preventive interventions. Creating a positive and supportive dental environment is essential to alleviate anxiety and encourage active participation, contributing to a more positive overall experience for the child [50].

Incorporating interactive elements into education sessions, such as interactive models or child-friendly videos, enhances understanding and engagement. Children are more likely to cooperate when they comprehend the purpose and benefits of preventive measures. This interactive approach not only educates but also makes the learning process enjoyable for the child [51].

Actively involving parents in the preventive process is crucial for sustained cooperation. Parents can reinforce oral hygiene practices at home and support their child during dental appointments, creating a collaborative approach to oral health care. This partnership enhances the continuity of preventive efforts beyond the dental office [52].

Consistent reinforcement of preventive practices is essential for sustained cooperation. Regular reminders, positive feedback, and age-appropriate incentives contribute to the establishment of oral health habits. This ongoing reinforcement reinforces the importance of preventive measures and encourages the child's active participation [35, 48].

Understanding individual patient behaviors and tailoring treatment strategies based on the child's temperament and comfort level are essential. Behavioral assessments help identify potential challenges and allow for personalized approaches to enhance cooperation. This individualized approach acknowledges the unique needs of each child, promoting a positive and cooperative attitude toward treatment measures for MIH.

### 8.2. Parental Education

Effectively communicating with parents and involving them in the preventive process is integral to the success of MIH preventive strategies. However, several challenges may impede seamless communication and collaboration [53].

Parents with limited dental knowledge may face challenges in understanding the complexities of MIH and the importance of preventive measures. Clear and accessible communication materials, possibly in multiple languages, can bridge this knowledge gap [42].

Busy lifestyles and time constraints may hinder parents' ability to prioritize and implement preventive measures effectively. Providing concise and practical guidance, along with flexible scheduling options for dental appointments, helps accommodate these challenges [35].

Cultural beliefs and perceptions about oral health may influence parental attitudes toward preventive measures. Culturally sensitive education that respects diverse perspectives fosters a collaborative and supportive relationship between dental professionals and parents [53].

Parents may harbor anxiety or concerns about their child's oral health, particularly in the context of MIH. Providing a platform for open communication, addressing parental concerns empathetically, and offering educational resources contribute to a shared decision-making process [54].

Utilizing technology, such as educational apps or virtual resources, facilitates parental education outside of the dental office. Interactive materials can enhance understanding and empower parents to actively participate in their child's preventive care [55].

Establishing a continuous line of communication with parents through follow-up appointments or informational sessions ensures that preventive measures are consistently reinforced. This ongoing dialog strengthens the partnership between dental professionals and parents [56].

In conclusion, successful treatment strategies for MIH hinge on overcoming challenges related to pediatric patient cooperation and effectively involving parents in the treatment process. Tailoring communication, utilizing behavioral management techniques, and fostering collaborative relationships contribute to a comprehensive approach that promotes oral health and minimizes the impact of MIH.

## 9. Resource Limitations

### 9.1. Access to Specialized Equipment

Limited access to specialized equipment and materials poses a significant challenge in the effective management of MIH. The nature of MIH treatment often requires specific tools and technologies that may not be readily available in all dental practices [16].

Accurate diagnosis of MIH relies on advanced diagnostic tools, such as optical coherence tomography [57, 58] or quantitative light-induced fluorescence [21]. The unavailability of these tools in some dental settings may hinder precise diagnosis and treatment planning.

Specialized restorative materials, such as resin infiltration agents or bioactive materials suitable for MIH-affected teeth, may not be universally accessible. The lack of access to these materials may limit treatment options and compromise the longevity of restorations [36].

The field of pediatric dentistry is witnessing advancements in technologies for MIH treatment, including novel remineralization agents [36]. However, the slow integration of these technologies into routine practice due to cost and training constraints hampers their widespread use.

Limited resources for continuing education and training on MIH-specific treatment protocols can further impede the adoption of best practices. Dental professionals may struggle to stay abreast of the latest developments, hindering the optimization of patient care [59].

Collaborating with other healthcare professionals, such as geneticists or pediatricians, may be challenging in settings where interdisciplinary communication channels are limited. This lack of collaboration can impact the comprehensive care required for cases of MIH associated with systemic conditions.

Addressing these challenges requires concerted efforts to improve infrastructure, provide targeted training, and promote the integration of specialized equipment and materials into routine dental practice. Advocacy for resource allocation in pediatric dentistry can contribute to better access to necessary tools.

### 9.2. Financial Constraints

Financial limitations significantly impact the feasibility of certain treatment options for MIH, affecting both dental practices and the families of affected children. The high costs associated with specialized restorative materials for MIH treatment can limit their utilization, particularly in resource-constrained environments. Dental practices may face financial barriers in procuring these materials, potentially compromising the quality of care provided to affected children [60].

The adoption of advanced diagnostic and treatment technologies, while beneficial for MIH management, requires substantial financial investments. Smaller practices or those in economically disadvantaged areas may struggle to acquire and maintain these technologies, limiting their ability to offer state-of-the-art MIH management to their patients.

Limited coverage by dental insurance plans for MIH-specific treatments can exacerbate financial constraints for families. The out-of-pocket expenses associated with MIH care may pose a barrier to accessing necessary treatments, impacting treatment adherence and overall outcomes for affected children [61].

Implementing preventive measures, such as professional fluoride applications or dental sealants, may be financially burdensome for some families. This financial strain can compromise the regularity of preventive interventions crucial for managing MIH, hindering the comprehensive care needed for affected pediatric patients [62].

Financial considerations may influence treatment planning decisions, potentially favoring less costly but suboptimal interventions (extraction). Dentists may need to navigate the delicate balance between providing effective care and considering the economic constraints of patients, emphasizing the need for a patient-centred approach [62, 63].

Addressing financial constraints requires a multifaceted approach, including advocacy for insurance coverage, financial assistance programs, and initiatives to make specialized materials and technologies more affordable and accessible. Additionally, fostering collaborative efforts between public and private sectors can contribute to reducing the economic barriers associated with managing MIH, ensuring equitable access to optimal care for all affected children.

In order to effectively manage the multifaceted challenges associated with MIH, it is essential to employ a strategic approach that combines diagnostic and therapeutic interventions.  Table 3 provides a comprehensive overview of the key challenges faced by dental practitioners in diagnosing and managing MIH, alongside corresponding interventions designed to mitigate these issues. This table highlights the complexities of MIH management, including challenges related to diagnosis, treatment planning, and patient compliance. By systematically outlining these challenges and the interventions available, this resource aims to facilitate informed decision-making and improve outcomes for pediatric patients affected by MIH.

## 10. Conclusion

The challenges associated with MIH in pediatric dentistry are multifaceted, encompassing various domains of clinical care, interdisciplinary collaboration, and psychosocial well-being.

1. Diagnostic complexity: The variability in MIH presentations and the overlap with other dental anomalies pose challenges in accurate and timely diagnosis. Standardized diagnostic criteria and advanced technologies are needed to enhance diagnostic precision.
2. Clinical management: Treatment planning complexities, patient cooperation, and the unpredictable progression of MIH lesions necessitate individualized approaches. Balancing age-appropriate interventions, psychosocial impacts, and long-term prognostic considerations is crucial for effective clinical management.
3. Long-term follow-up: Consistent follow-up care is hindered by patient adherence, financial constraints, and the need for treatment plan adjustments over time. Strategies such as patient education, financial assistance programs, and technological solutions are essential to ensure ongoing monitoring and care.
4. Psychosocial impact: Esthetic concerns, potential teasing or bullying, and communication challenges contribute to the psychosocial impact of MIH. A holistic approach involving effective communication, cultural sensitivity, and collaboration with mental health professionals is crucial for addressing psychosocial challenges.
5. Resource limitations: Limited access to specialized equipment, materials, and continuing education hampers optimal care delivery. Advocacy for resource allocation, financial assistance programs, and enhanced educational initiatives can address these limitations.

In overcoming these challenges, the importance of ongoing research, education, and collaboration cannot be overstated. Continued research efforts are needed to unravel the complexities of MIH etiology, refine diagnostic criteria, and advance treatment modalities. Education initiatives must target both dental professionals and the broader healthcare community, fostering interdisciplinary collaboration and promoting awareness of the psychosocial aspects of MIH.

Collaboration, not only within the dental field but also with other healthcare disciplines, is fundamental for a comprehensive and patient-centred approach. Interdisciplinary care plans, effective communication channels, and shared decision-making processes can enhance the quality of care for pediatric patients with MIH.

In conclusion, the challenges posed by MIH demand a concerted and collaborative effort from the dental community, healthcare professionals, researchers, and educators. By addressing these challenges collectively, the pediatric dental landscape can evolve toward improved diagnostic accuracy, treatment efficacy, and enhanced psychosocial well-being for children affected by MIH.

## Figures and Tables

**Table 1:** Search strings for PubMed, Scopus, and Google Scholar databases.

Database	Search strings
PubMed	((“molar hypomineralization”[MeSH Terms] OR (“molar”[All Fields] AND “hypomineralization”[All Fields]) OR “molar hypomineralization”[All Fields] OR (“molar”[All Fields] AND “incisor”[All Fields] AND “hypomineralization”[All Fields]) OR “molar incisor hypomineralization”[All Fields]) AND (“aetiologie”[All Fields] OR “aetiologies”[All Fields] OR “aetiology”[All Fields] OR “etiologies”[All Fields] OR “etiology”[MeSH Subheading] OR “etiology”[All Fields] OR “causality”[MeSH Terms] OR “causality”[All Fields] OR (“diagnosable”[All Fields] OR “diagnosi”[All Fields] OR “diagnosis”[MeSH Terms] OR “diagnosis”[All Fields] OR “diagnose”[All Fields] OR “diagnosed”[All Fields] OR “diagnoses”[All Fields] OR “diagnosing”[All Fields] OR “diagnosis”[MeSH Subheading]) OR (“therapeutics”[MeSH Terms] OR “therapeutics”[All Fields] OR “treatments”[All Fields] OR “therapy”[MeSH Subheading] OR “therapy”[All Fields] OR “treatment”[All Fields] OR “treatments”[All Fields]) OR (“prognosis”[MeSH Terms] OR “prognosis”[All Fields] OR “prognoses”[All Fields]))) (“2013/01/01”[PDat]: “2023/12/01”[PDat])

Scopus	TITLE-ABS-KEY (Molar Incisor Hypomineralization AND Children) AND TITLE-ABS-KEY (etiology OR diagnosis OR treatment OR prognosis) Scopus—Document search results

Google Scholar	“molar incisor hypomineralization” AND (etiology OR diagnosis OR treatment OR prognosis)

**Table 2:** Prevalence of molar incisor hypomineralization (MIH) across different regions and populations.

Region	Prevalence range (%)	Study references
Global	13.5 (mean prevalence)	Lopes et al. [14, 15]
Middle East	2.3–40.7	Bukhari et al. [12]
Europe	3–37	Lygidakis et al. [16]
South America	12–20	Jalevik [17]; Lopes et al. [14, 15]
Australia and New Zealand	6.3–22.1	Elfrink et al. [10]
Asia	9.5–44	Pentapati, Yeturu, and Siddiq [11]
Africa	2.3–14.9	Kassebaum et al. [7]

**Table 3:** Summary of challenges and interventions in the diagnosis and management of molar incisor hypomineralization (MIH).

Challenges	Interventions
Diagnosis	
Variability in clinical presentation	Use of standardized diagnostic criteria (EAPD's criteria) for more consistent and accurate identification
Early detection of subtle enamel defects	Advanced diagnostic tools such as reveal fluorescence dental loupes for identifying early lesions
Differentiating MIH from other enamel defects	Detailed clinical examination and comparison with conditions like fluorosis and enamel hypoplasia
Management	
Esthetic concerns in visible anterior teeth	Use of minimally invasive treatments like resin infiltration or composite bonding to restore esthetics
Sever hypersensitivity in affected teeth	Application of desensitizing agents such as fluoride varnish, casein phosphopeptide-amorphous calcium phosphate (CPP-ACP), and use of SDF to manage sensitivity
Risk of caries development in weakened enamel	Preventive measures including fluoride varnish, regular professional cleanings, and dietary guidance to reduce caries risk
Limited cooperation from pediatric patients	Employing behavioral management techniques, such as tell–show–do, or sedation in severe cases to enhance compliance during treatment
Long-term restorations in compromised teeth	Use of glass ionomer cement or composite resin restorations in mild cases or stainless steel crowns in more severe cases to maintain tooth structure
Need for extraction in cases of severe damage	Strategic extractions, followed by space maintainers or orthodontic evaluations, to manage occlusion and future dental development
Psychosocial impact of visible MIH lesion	Early esthetic treatment, open communication with the child and family, and referral to mental health professionals if needed
Resource limitation in some clinical settings	Focus on basic preventive care (oral hygiene education, fluoride treatments) and use of affordable materials like glass ionomer

## Data Availability

The data that support the findings of this study are available from the corresponding author upon reasonable request.
